# Recurrence of Gastric Mucosa-associated Lymphoid Tissue Lymphoma in the Pleura: A Case Report

**DOI:** 10.7759/cureus.5150

**Published:** 2019-07-16

**Authors:** Edward Charbek, Gebran Khneizer, Karen Moser, Setu Patolia

**Affiliations:** 1 Internal Medicine, Saint Louis University School of Medicine, St. Louis, USA; 2 Internal Medicine, Indiana University Hospital, Indianapolis, USA; 3 Pathology, Saint Louis University School of Medicine, St. Louis, USA

**Keywords:** malt lymphoma, pleural disease

## Abstract

Mucosa-associated lymphoid tissue (MALT) lymphoma is classified as marginal zone lymphoma, a form of low-grade malignant B-cell non-Hodgkin’s lymphoma. It affects the gastrointestinal tract with lung and pleural involvement considered to be rare. We describe a case of a 71-year-old man with a history of MALT lymphoma in remission who presented with dyspnea due to pleural effusion. Pleural fluid flowcytometry analysis showed monotypic B-cell population that expressed cluster of differentiation (CD)19, CD20, CD22, and kappa surface light chains. Medical pleuroscopy and pleural biopsy showed fibroadipose tissue with poorly defined lymphoid aggregates displaying a so-called “monocytoid” appearance, a histologic finding typical of marginal zone lymphoma. The patient underwent pleurodesis and achieved resolution of pleural effusion; however, the patient developed several complications and was discharged on home hospice.

## Introduction

Mucosa-associated lymphoid tissue (MALT) lymphoma is a low-grade non-Hodgkin's lymphoma (NHL) which frequently affects the gastric mucosa. MALT lymphomas are uncommon, accounting for 5% of all NHL [1]. Such malignancy is associated with autoimmune disorders or chronic inflammation which in most cases is caused by Helicobacter pylori (H. pylori) infection. Ample literature has described the occurrence of primary pulmonary lymphoma, pleural lymphoma, or recurrence of lymphomas in the pleura [2], but gastric MALT lymphoma recurrence in the pleura has not been reported. We present a case of gastric MALT lymphoma recurrence in the pleural cavity after eradication of H. pylori.

## Case presentation

A 71-year-old male with a history of gastric MALT lymphoma status post H. pylori treatment and confirmed eradication presented with progressively worsening shortness of breath and productive cough. He had noticed the dyspnea two months prior to presentation and reported exertion as the main trigger. There was no nausea, vomiting, diarrhea, constipation, fever, chest pain, or appetite changes. However, he reported a 13-pound unintentional weight loss over six months. The patient has a past medical history that is significant for chronic obstructive pulmonary disease (COPD) with chronic respiratory failure requiring supplemental home oxygen, heart failure with preserved ejection fraction, and deep vein thrombosis. The patient is an active smoker with more than 50 pack-year tobacco use. He has no history or risk factors of tuberculosis. There was no history of alcohol use or illicit drug use. Medications prior to admission were furosemide, lisinopril, pantoprazole, tiotropium bromide monohydrate inhaler, and albuterol inhaler.

The patient was diagnosed with gastric MALT a year prior to the current presentation and underwent eradication of H. pylori infection with amoxicillin, clarithromycin with a proton pump inhibitor with resolution of the gastric tumor.

**Figure 1:**
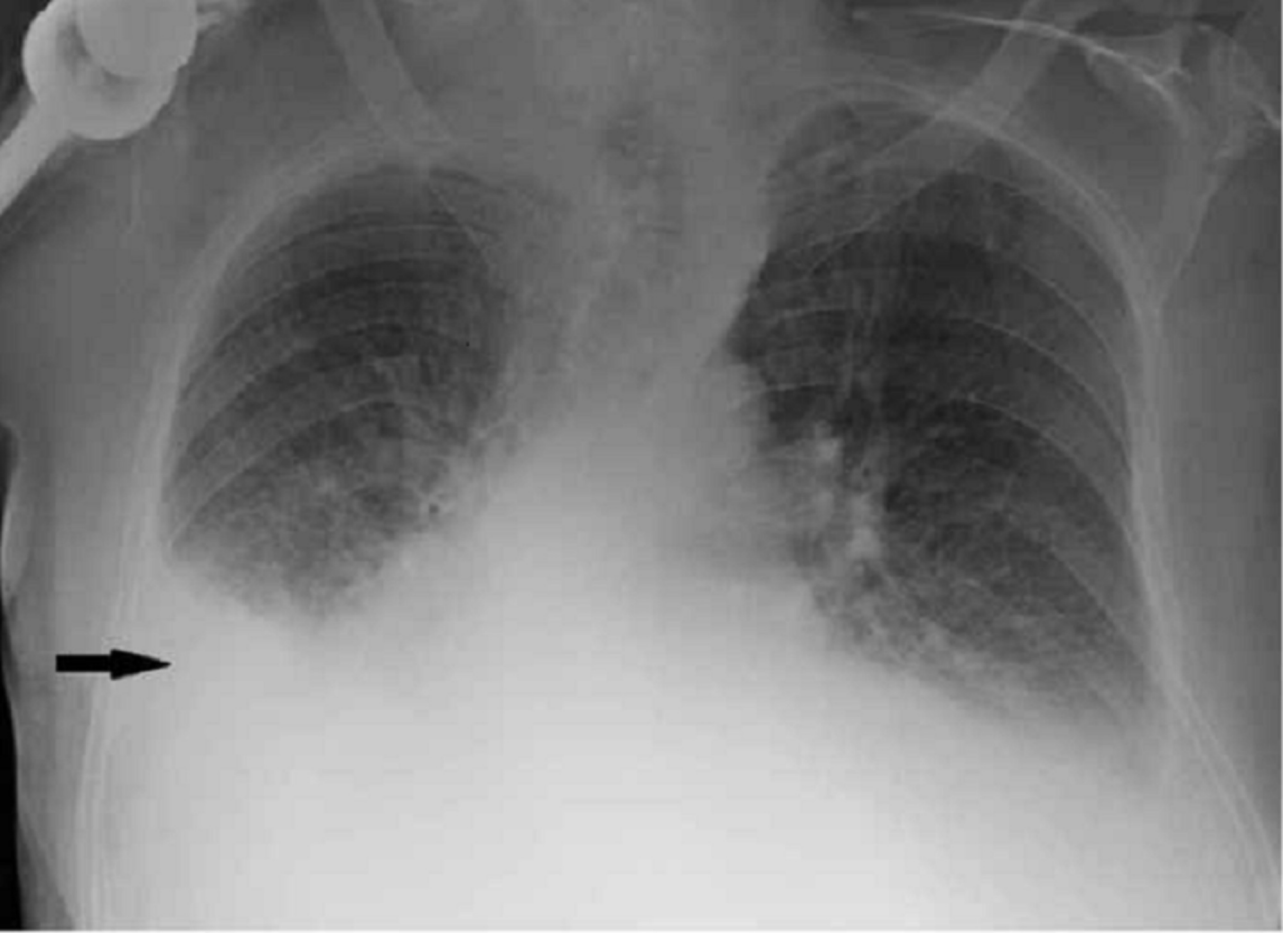
Posteroanterior radiograph showing moderate size right sided pleural effusion (arrow)

**Figure 2:**
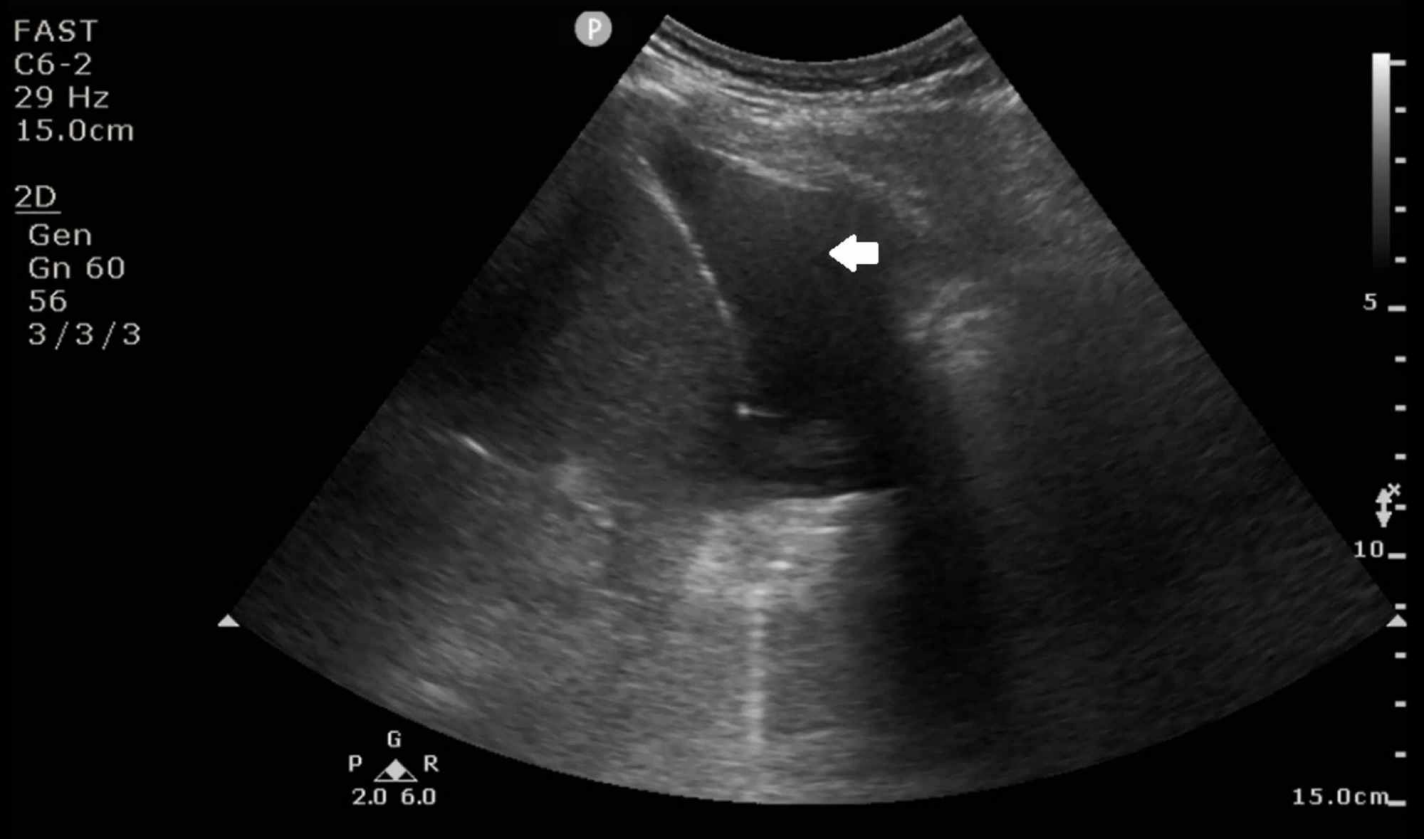
Ultrasound showing the moderate size right sided pleural effusion (arrow)

On physical examination, his temperature was 36.8 °C, his blood pressure was 121/72 mm Hg, his pulse rate was 78 beats per minute, and his respiratory rate was 18 breaths per minute. He had scattered wheezing and decreased breath sounds over the right lung base. The rest of the examination was unremarkable. A chest X-ray with posteroanterior and lateral views revealed a large right pleural effusion which was confirmed on thoracic ultrasonography (Figures 1-2).

The patient underwent thoracentesis with the removal of 750 milliliters of exudative fluid based on Light’s criteria (Table 1). Cytologic assessment of the pleural fluid revealed 90% lymphocytes with no malignant cells. The pleural effusion recurred within three days and the pulmonary team elected to pursue medical pleuroscopy, pleurodesis, and pleural biopsy for further evaluation and prevention of further recurrence. The procedure was followed by chest tube placement which was removed four days later.

**Table 1:** Pleural fluid analysis LDH: Lactate dehydrogenase; WBC: White blood cells; RBC: Red blood cells.

Parameter(Unit)	Result	Reference Range
Color	yellow	----
WBC(/uL)	551	Not established
RBC(/uL)	4000	Not established
Lymphocytes(%)	90	Not established
Monocytes(%)	6	Not established
Glucose (mg/dL)	126	Not established
LDH( Units/L)*	105	Not established
Protein(g/dL)	4.2	Not established

**Figure 3:**
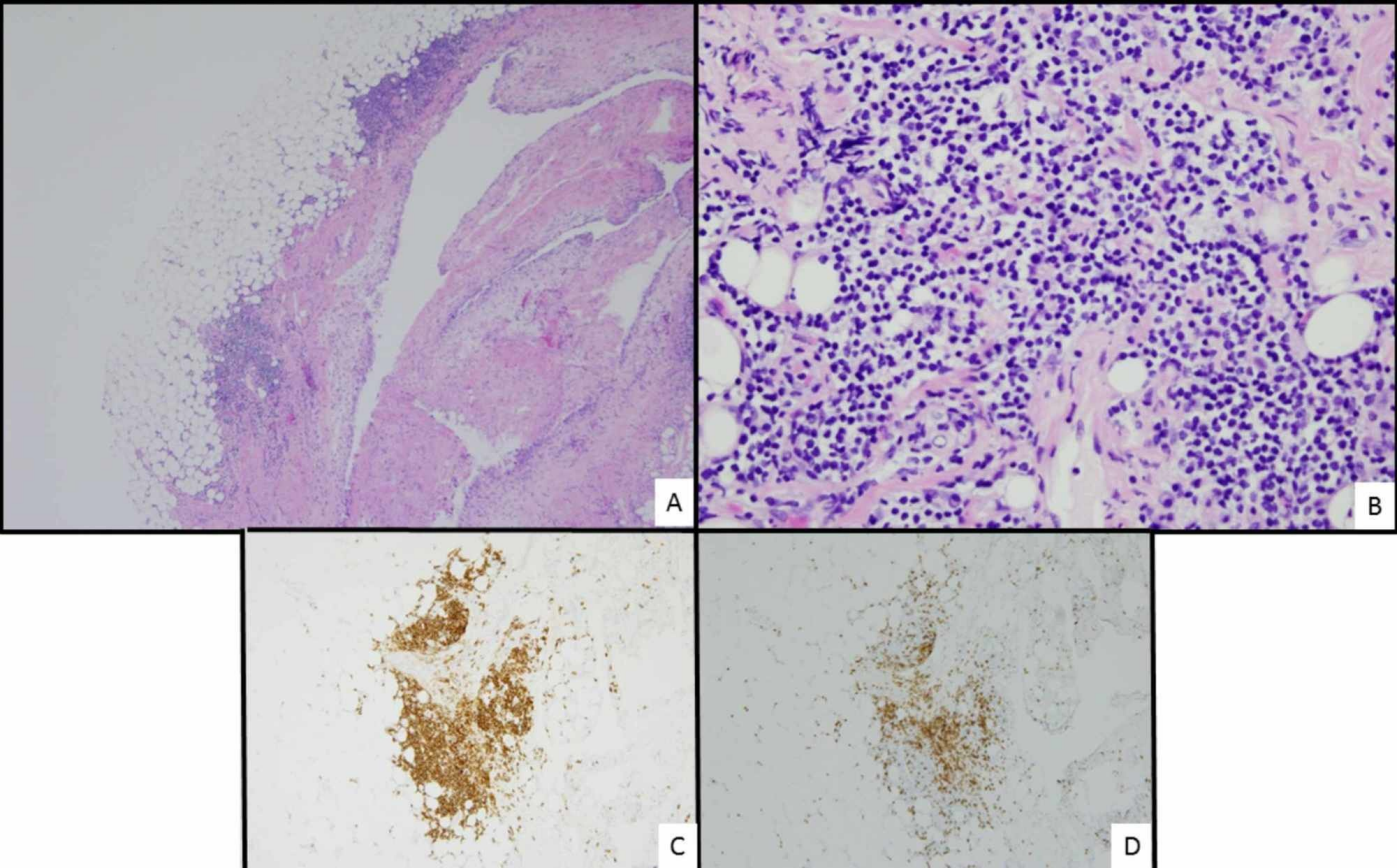
Biopsy of the right pleura A) Low power view of pleura biopsy showing aggregates of small lymphocytes (hematoxylin and eosin (H&E), 4x). B) High power view of pleura biopsy demonstrating that the lymphocytes are small and mature in appearance, with focal monocytoid features (H&E, 40x). C) A majority of the lymphocytes are B-cells, staining positively for CD20 (CD20 immunohistochemistry, 10x). D) The lymphoid aggregates contain few small T-cells, staining positively for CD3 (CD3 immunohistochemistry, 10x).

The biopsy of the right pleura demonstrated fibroadipose tissue with multiple poorly defined lymphoid aggregates (Figure 3). The lymphocytes within these aggregates were small in size, with predominantly round nuclear contours, condensed chromatin, no identifiable nucleoli, and moderate amounts of pale-staining cytoplasm. Focally, the lymphocytes displayed a so-called “monocytoid” appearance, a histologic finding typical of marginal zone lymphoma. By immunohistochemistry, the majority of the lymphocytes were cluster of differentiation (CD)20-positive B-cells, with few infiltrating small CD3-positive T-cells. Flow cytometric immunophenotyping performed on a concurrent pleural fluid specimen identified a monotypic B-cell population that expressed CD19, CD20, CD22, and kappa surface light chains, but not CD5 or CD10. Given the patient’s known history of gastric extranodal marginal zone lymphoma, the morphologic and immunophenotypic findings in the pleural biopsy and pleural fluid were interpreted as representing pleural involvement by extranodal MALT lymphoma.

**Figure 4:**
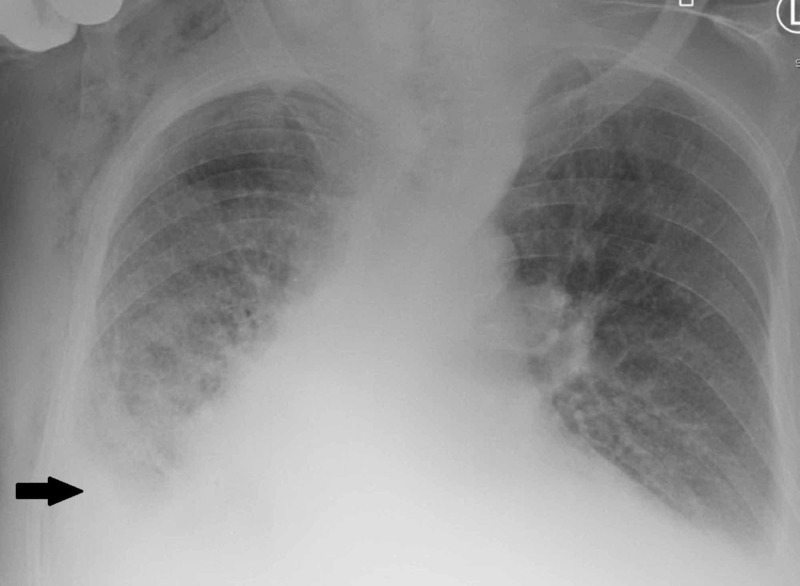
Posteroanterior radiograph showing decrease of right sided pleural effusion volume after pleurodesis (arrow)

The patient responded to pleurodesis with minimal recurrence of the pleural effusion after chest tube removal and improvement in dyspnea (Figure 4). However, the hospital course was complicated with a complete heart block. In light of advanced disease and multiple comorbidities, the patient declined any intervention and elected to pursue comfort care measures. He was discharged to home with hospice care.

## Discussion

MALT lymphoma, a form of indolent NHL, can arise from the majority of mucosal linings such as bronchus-associated lymphoid tissue (BALT) and gut-associated lymphoid tissue (GALT). MALT lymphoma comprises 5% of all NHL and gastric involvement constitutes about 85% of all MALT lymphomas [3]. Since this malignancy has been commonly associated with chronic inflammation, infectious agents such as H. pylori are considered prominent risk factors in the pathogenesis of gastric MALT lymphoma. Gastric MALT lymphoma usually manifests as a low-grade lymphoma with a minority of cases transitioning into high-grade malignancies. Eradication of H. pylori infection with standard therapy leads to complete remission of the lymphoma in around 80% of cases [4].

Lymphomas of the thorax include a variety of histologic subtypes: MALT lymphoma, diffuse large B-cell lymphoma, Burkitt lymphoma, mantle cell lymphoma, and follicular lymphoma. MALT lymphoma is the most common primary pulmonary lymphoma. Tobacco exposure is considered a risk factor with 45% of pulmonary MALT lymphoma patients have a history of smoking [5]. Collagen vascular diseases are seen in approximately 10% to 29% of patients with MALT lymphoma, with Sjogren’s syndrome being the most common disease associated with MALT lymphoma [6]. Pulmonary MALT lymphoma has an overall favorable prognosis with a five-year survival rate of greater than 80% and a median survival greater than 10 years [7].

There exists large literature on primary pulmonary lymphoma [2] including primary pleural MALT lymphoma occurrence [8], but reports of the gastric MALT lymphoma recurrence to the lung are limited. While the recurrence of gastric MALT lymphoma to the endobronchial tree has been described [9], there is no report of the pleura as the primary site of recurrence. This could be due to the rarity of the condition or the need for pleural biopsy to confirm the diagnosis. In our patient, the initial pleural fluid flow cytometry was not confirmatory for MALT lymphoma; thus, this mandated pleural biopsy. To our knowledge, this is the first case of recurrence of gastric MALT lymphoma to the pleura. Therapeutic approaches aim for symptomatic relief and are similar to the management of other malignant effusions such as palliative chemotherapy, chemical or surgical pleurodesis, and indwelling pleural catheters [10].

This case also describes how chemical pleurodesis can be used as a palliative option in pleural lymphomas.

Our case highlights two important clinical points. First, recurrence of such lymphoma to the pleura should be included in the differential diagnosis of patients presenting with recurrent pleural effusion, particularly those with prior history of gastric MALT lymphoma. Second, pursuing invasive procedure to obtain tissue sample might prove to be necessary for diagnosis as symptoms, radiographic findings, and conventional studies performed on the pleural fluid are usually nonspecific and may not reveal the final diagnosis.

## Conclusions

In patients with recurrent pleural effusion and a prior history of gastric MALT lymphoma, clinicians need to maintain a high index of suspicion for recurrence of such neoplasm in the pleural cavity.
